# Method of Manipulating Gold as Practised Fifty Years Ago

**Published:** 1894-10

**Authors:** F. B. Smith

**Affiliations:** New York City


### METHOD OF MANIPULATING GOLD AS PRACTISED FIFTY YEARS AGO.[1]

BY F. B. SMITH, M.D., NEW YORK CITY.

The method of manipulating the gold as practised by Dr. Eleazer Parmly in filling teeth fifty years ago may have had some points of merit that possibly may have been overlooked, and may be unknown to some of you present this evening.

Having seen stoppings that were put in by him and had remained preserving the teeth forty and fifty years is sufficient proof that his method of filling teeth was not without merit, worthy of our consideration and careful investigation, and possibly the points of the pluggers used by him were well adapted to that style of filling, and even better, perhaps, than any of the many varieties that have been used since.

So long as the object of filling teeth is or should be to preserve the teeth for the longest possible time, the best means how to accomplish that end should ever be the ardent desire and never-ending study of every dentist, and when we disregard the laws of nature and those that aid us in manipulating gold we surely must be on the wrong track.

Dr. Eleazer Parmly took gold in square form, laying three or four pieces one upon the other, according to the size of the cavity to be filled, with the plugger point in the centre, and giving the gold a twist at the end of the plugger point, and placing it in the cavity, pressing it in and around to the walls, then turning the gold above the cavity over into the centre of the gold, leaving a well-turned edge all around the margin and a well-formed cavity in the gold; then another layer of gold arranged the same as was the first, and packed in the same way, and so on until the cavity is full, and finishing the stopping in the centre of the gold, always carrying the point of the plugger in the same direction, the same as if turning in a screw from first to last, twisting as it were the gold down into the cavity. In this way of packing gold the

---

[1] Read before the New York Odontological Society, June 19, 1894.

plugger never comes in contact with the wall of the cavity, and there is less danger of bruising or breaking down the canals or tubules of the bone, and the cut-under shape of the cavity is retained in each layer of the gold as it was put in, thus securing the last piece of gold as securely as the first.

Some years ago when operating for one of Dr. Eleazer Parmly's former patients, I found a large crown stopping in a lower molar tooth that the lady said was put in by Dr. Parmly some twenty years before; it apparently needed renewing; the gold was so soft on pressure with the instrument it led me to suspect considerable decay under the gold. When I took the gold out the cavity was as perfect and free from decay as it was the day it was formed, as round and smooth as a bur-head could make it; the cut-under was hardly perceptible. I refilled it as it was.

Some three years ago I saw a tin stopping put in by Dr. E. Parmly when his office was in Park Place, still remaining and preserving the tooth. The reason for filling it with tin, the lady said, was that the nerve was so nearly exposed the doctor considered it a safer material than gold, as it required less pressure in putting it in.

